# Brevinin-2R-linked polyethylenimine as a promising hybrid nano-gene-delivery vector 

**DOI:** 10.22038/ijbms.2019.37125.8842

**Published:** 2019-09

**Authors:** Fatemeh Zohrab, Ahmad Asoodeh, Amin Jalili, Majid Darroudi, Reza Kazemi Oskuee

**Affiliations:** 1 Department of Medical Biotechnology and Nanotechnology, Faculty of Medicine, Mashhad University of Medical Sciences, Mashhad, Iran; 2 Department of Chemistry, Faculty of Science, Ferdowsi University of Mashhad, Mashhad, Iran; 3 Nuclear Medicine Research Center, Mashhad University of Medical Sciences, Mashhad, Iran; 4 Targeted Drug Delivery Research Center, Institute of Pharmaceutical Technology, Mashhad University of Medical Sciences, Mashhad, Iran

**Keywords:** Brevinin 2R, Cell penetrating peptide, Gene delivery Polyethyleneimine, Vector

## Abstract

**Objective(s):**

Polyethylenimine (PEI) is one of the most widely used polymers in gene delivery. The aim of this study was to modify PEI by replacing some of its primary amines with Brevinin 2R (BR-2R) peptide in order to increase the efficiency of gene delivery.

**Materials and Methods:**

Polyethylenimine was modified by BR-2R peptide by two different approaches; A) conjugation methods including (І) using succinimidyl 3-(2-pyridyldithio) propionate (SPDP), (П) EDC/NHS protocol and (ПІ) EDC/NHS+6-bromohexanoic acid protocol, and B) physical interaction method. The modified polymers were characterized for their ability of plasmid condensation, number of primary amines, size and zeta potential. The transfection efficiency and cytotoxicity were evaluated on HEK293, L929, WEHI164 and Neuro2A cell lines by green fluorescent protein (GFP)-based plasmid (pGFP) reporter gene and viability assays, respectively. Apoptosis induction ability was also evaluated via PI/Annexin V assay.

**Results:**

Polyplex had size and zeta potential between 200-270 nm and +21.5- +28.4 mV, respectively. All vectors were able to condense plasmid DNA in C/P=4 (carrier-plasmid ratio). Transfection results on the Neuro2A cell line showed that the vector containing the BR-2R peptide, which was synthesized using EDC-NHS protocol had the best transfection efficiency.

**Conclusion:**

Our results showed that conjugation of Brevinin 2R as cell penetrating peptide to polyethyleneimine could enhance the transfection ability of the polymer.

## Introduction

Although gene transfer efficiency by viral carriers is much higher than other types of delivery approaches, some limitations such as immunogenicity, limited capacity, and carcinogenicity of viral carriers have led researchers into focusing on the application of two major non-viral carrier’s types (1, 2), which involve cationic polymers (3) and cationic lipids (4). As a cationic polymer, polyethyleneimine (PEI) has been used as *in vitro* and *in vivo* gene-delivery carrier since 1995 (5). PEI consists of repetitive units of CH2-CH2-NH and has been used in two branching (b-PEI) and linear (l-PEI) forms. PEI polymer has toxic effect that depends on the concentration and molecular weight. The most suitable molecular weight of PEI for transfection has been reported to be 25 kDa (6).

In comparison to some other polymers such as PLL, the proton-sponge effect of PEI (caused by the presence of a large number of amine groups in the chemical structure of the polymer) could facilitate endosomal escape and thus, increase the transfection efficiency of the polymer (7-11). In addition, the high cationic charge density of PEI can affect the polyplex size (12). However, this high positive charge of PEI surface leads to some unwanted interactions with the cell membrane which causes to disruption of cell membrane (13). In spite of selective permeability of cell membrane, which is essential for cell function and its survival, the membrane has become a daunting barrier for successful cellular penetration of gene delivery vehicles. The employment of low molecular weight pro-drugs, liposomes, micro, and nanoparticles has been developed for this purpose; however, the use of cell penetrating peptides (CPPs) is a new approach that has been studied. The CPP is a short peptide sequence that contains less than 35 amino acid residues and is capable of passing through the cell membrane and deliver the cargo into the cell. Although, the precise cell penetration mechanism of these peptides have not been clearly specified, the number of CPP amino acids, vector molecular weight, and cell type, as well as the incubation conditions such as temperature and time, seem to have a significant effect on the cell penetration pathway (14, 15). However, the two main penetrating mechanisms related to these peptides are energy dependent endocytosis and energy independent direct penetration (16). Lysine and arginine amino acids can cause positive charges in the peptides with cell penetration capabilities (17). In fact, the electrostatic interactions between the negative charges of the cell membrane and these positive charges of CPP can extend the connection to the cell membrane and thus, increase the amount of penetration into the cell. Brevinin 2R (BR-2R) is an amphiphilic peptide which has been first extracted from amphibian skin called Rana ridibunda that is composed of 25 amino acids (KLKNFAKGVAQSLLNKASCKLSGQC), with a di-sulfide bond located at the hydrophobic end of the peptide (Between amino acids 19 and 25). The peculiar features of this peptide are various including anti-angiogenesis, semi-apoptotic in cancer cells, and extensive antimicrobial effects (18-21). Considering the advantages and disadvantages of both viral and non-viral gene delivery vectors, the concept of synthetic viruses or hybrid vectors has developed (22). Hybrid vectors are the new generation of viral vectors in which viruses or viral peptides combined into the different synthetic vector such as the cationic and amphiphilic-based vectors. The main purpose for developing hybrid vector is improving the transfection efficiency of non-viral vectors and decreasing the immune system stimulation of viral vectors (23-25). Our approach in this study was to use low molecular-weight branched PEI polymer (PEI 10 kDa) to avoid toxicity problems appears with the use of 25 kDa PEI. To overcome the obstacles of the utilization of low molecular weight polymers such as low cellular penetration and transfection efficacy, 10 kDa PEI was conjugated to BR-2R as a novel cell penetrating enhancer peptide.

## Materials and Methods

The normal cell lines [HEK293 (ATCC CRL-1573) & L929 (ATCC CCL-1)] and the cancerous cell lines [WEHI164 (ATCC CRL-1702) & Neuro2A (ATCC CCL-131)] were purchased from Pasteur institute of Iran. Peptide BR-2R has been obtained from GL Biochem, China. The 6-bromohexanoic acid, 2,4,6-trinitrobenzene sulfonic acid (TNBS), and methylthiazoletetrazolium [3-(4,5-dimethylthiazol-2-yl)-2,5-diphenyl tetrazolium bromide] have been acquired from Sigma–Aldrich (Munich, Germany). Dulbecco’s modified eagle’s medium (DMEM) and fetal bovine serum (FBS) have been bought from GIBCO (Gaithersburg, USA). Dialyses were performed through the utilization of Spectra/Por dialysis membranes (Spectrum Laboratories, Houston, USA). 1-Ethyl-3-(3-dimethylaminopropyl) carbodiimide (EDC) and N-hydroxysuccinimide (NHS) were obtained from Sigma-Aldrich (Munich, Germany). Branched polyethyleneimine (b-PEI 10 kDa) has been procured from Polysciences Inc. (Warrington, PA, USA). HEPES and N-succinimidyl 3-(2-pyridyldithio) propionate (SPDP) have been purchased from Sigma-Aldrich (Munich, Germany) and dimethyl sulfoxide (DMSO) and MTT dye 3-(4,5-dimethylthiazol-2-yl)-2,5-diphenyltetrazoliumbromide have been ordered from Sigma-Aldrich (Munich, Germany). The pEGFP-N1 coding for EGFP DNA has been obtained from Invitrogen (Clontech, Palo Alto, CA, USA).

***Conjugation of peptide to the PEI***

*I. SPDP conjugation method*

A. Synthesis of 3-(2-pyridyldithiol) propionate-modified PEI

**Figure 1:**
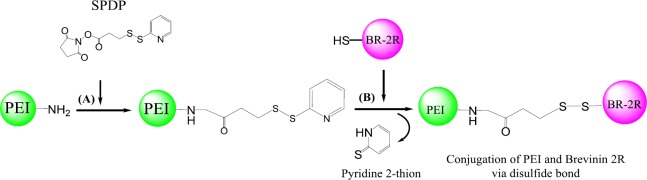
Schematic synthesis of brevinin 2R (BR-2R)-linked polyethylenimine (PEI) by SPDP conjugation method

PEI (10 kDa) was mixed with SPDP (3.8 μmol; 1.19 mg) as cross-linking reagent dissolved in 2 ml HEPES buffer (20 mM, pH 8.0, containing NaCl 0.35 M). After 2 hr stirring at room temperature under argon gas, the PEI with N-succinimidyl 3-(2-pyridyldithio) propionate linkers (PEI-SPDP) was purified by dialysis with 3-kDa molecular weight cutoff membranes. The degree of modification with SPDP linker was ascertained by spectrophotometrically absorbance assay of released pyridine-2-thione in the medium of its reducing agent (dithiothreitol) at 343 nm. As the control assay, 10 µl of the sample was added to 140 µl water and the blank absorbance was measured at 343 nm. The primary amine contents of pyridyldithiol-activated PEI were discovered by utilizing the trinitrobenzenesulfonic acid (TNBS) assay at 405 nm, as it has been explained previously (26). At the end, the samples containing PEI-SPDP were pooled, aliquots, flash frozen and stored at − 20^ °^C (Figure 1).

B. Synthesis of BR-2R-conjugated PEI

Brevinin 2R (BR-2R) peptide (1.38 µmol; 4 mg) was dissolved in 400 µl deionized water and mixed with the activated PEI-SPDP by a rapid vortex for 30 min followed by dialysis via 3-kDa molecular weight cutoff membrane. After 4 hr incubation at room temperature, the concentration of released pyridine-2-thion was calculated by measuring the absorbance at 343 nm in order to determine the extent of peptide conjugation. The degree of unmodified amine groups was determined by the TNBS test at 405 nm. The preceding solutions of the peptide conjugates were flash-frozen in aliquots and kept at a temperature of - 20 ^°^C until required to be used (26).

*II. EDC/NHS conjugation method*

**Figure 2:**
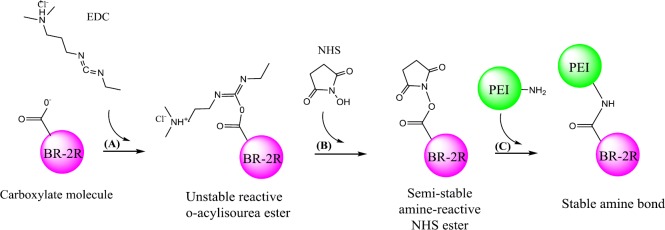
Schematic synthesis of brevinin 2R (BR-2R)-linked polyethylenimine (PEI) by EDC/NHS conjugation method

The peptide bond was created between PEI and peptide through the usage of EDC/NHS method (Figure 2). Briefly, in order to activate the terminal carboxylic acid of BR-2R peptide, 1.0 mole equivalent of EDC/NHS solution was added to BR-2R solution and stirred for 30 min at ambient temperature. Then, aqueous solution of carboxyalkylated BR-2R peptide was mixed slowly with excess solution of 10 kDa PEI and incubated for 20 min at room temperature. Finally, the reaction mixture was purified using 10 kDa cutoff Spectra/Por dialysis tubing against four changes of time (3, 24, 48, and 72 hr). The dialyzed solution was lyophilized and stored at -20 ^°^C (27).

*III. EDC/NHS+6-bromohexanoic acid conjugation method*

**Figure 3:**
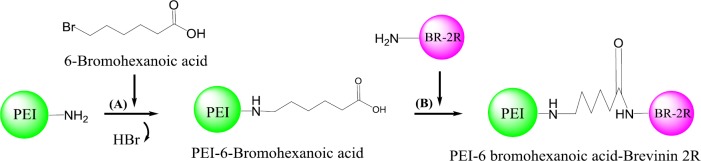
Schematic synthesis of brevinin 2R (BR-2R)-linked polyethylenimine (PEI) by 6-bromohexanoic acid linker

6-bromohexanoic acid was dissolved in DMF and the solution was added drop-wisely to an stirring solution of PEI in DMF at room temperature over the 1 hr. The reaction was permitted to continue for a period of 24 hr. The crude products were dialyzed in 10 kDa cut-off Spectra/Por dialysis tubing once against NaCl (0.25 M) and then against water to discard the unreacted alkylating agents followed by lyophilization (28). Activated BR-2R peptide (prepared by EDC/NHS protocol) was added to the PEI-6-bromohexanoic acid to form PEI-6-bromohexanoic acid- BR-2R (Figure 3).

***Physical interaction method***

For physical interaction of BR-2R and PEI 10 kDa, different molar ratios of PEI solution (with the similar ratio in conjugation methods), was added to the peptide solution (1 mg/ml) and stirred for 2 hr at room temperature.

***Determination of the degree of grafting using 2,4,6-trinitrobenzenesulfonic acid***

The primary amine content of unmodified-PEI and modified-PEI was determined by utilizing the 2, 4, 6-trinitrobenzenesulfonic acid (TNBS). Briefly, standard PEI solutions were serially diluted in 0.1 M sodium tetraborate (final volume of 100 µl). To each well of 96-well plate, 2.5 µl of TNBS (75 nmol; 22 µg; diluted in deionized water) was added. After 10 min the absorption of colored trinitrophenylated derivatives (formed by the reaction between TNBS and primary amino groups of samples) was measured at 405 nm using a microplate reader at room temperature (26).

***Gel retardation assay***

The complexation capacity of the vectors was investigated through the employment of an agarose gel retardation assay. Polyplexes were prepared as it has been specified before and set down into a 1% (w/v) agarose gel in TBE buffer (trizmabase 10.8 g, boric acid 5.5 g, disodium EDTA 0.75 g, and water 1.00 l) containing green viewer dye. Electrophoresis was performed at 80 V for the duration of 40 min and was assessed under ultraviolet light. Polyplexes of modified PEI, BR-2R peptide and unmodified PEI 10 kDa with C/P ratios of 0.5, 1.0, 2.0, 4.0, and 6.0 were prepared and used for gel retardation assay.

***Particles size and zeta potential measurements***

The particle size and zeta potential of polyplexes were measured through the usage of a dynamic light scattering (DLS) and laser doppler velocimetry (Malvern Nano ZS, Malvern Instruments, Malvern, UK), respectively. Polymer/ Plasmid DNA (pDNA) complexes have been formed in HBG buffer at C/P ratios of 4.0 by mixing equal volumes of carries and plasmid solutions. The data have reported as mean±standard deviation (SD) of 3 measurements. 

***Cell culture***

HEK293, L929, WEHI164 and Neuro2A cell lines were cultured in Dulbecco’s modified Eagle’s medium (1.0 g/l glucose, 2 mM glutamine) that was formerly augmented with 10% FBS, streptomycin at 100 μg/ml, and penicillin at 100 U/ml. All of the involved cells were incubated at a temperature of 37 ^°^C in a humidified 5% CO_2_ atmosphere.

***In vitro transfection assay***

The cells (including HEK293, L929, WEHI164 and Neuro2A) were seeded at a density of 10,000 cell/well 24 hr prior to the transfection experiment. Polyplexes were prepared and added to the cells in three various C/P ratios in order to optimize the transfection circumstances. After 4 hr, the medium was aspirated off and exchanged with fresh DMEM medium containing serum followed by incubation for another 24 hr at 37 ^°^C.

***Cell viability assay***

*In vitro* polyplexes cytotoxicity was determined through the 3-(4,5-dimethylthiazol-2-yl)-2,5-diphenyltetrazoliumbromide (MTT) assay on HEK293, L929, WEHI164 and Neuro2A cells in various C/P ratio. The cells were seeded at a density of 10,000 cells/well in 96-well micro assay plates and incubated for a period of 24 hr prior to the examination. The polyplexes were added to the cells and incubated in a situation similar to that of the transfection protocole. After 24 hr, 10 μl of MTT solution (5 mg/ml in PBS) was added to each well followed by incubation at temperature of 37 ^°^C for 2 hr. Later on, the medium was aspirated off and 100 μl of DMSO was added to liquefy the formazan crystal that has been created by the live cells. Final absorbance was measured at 540 and 630 nm (Reference wavelengths). The metabolic activity of the polyplex-treated cells was considered in relative to untreated cell a control (Which was considered as 100% metabolic activity).

***Apoptosis induction assay***

Annexin V-FITC apoptosis detection assay was utilized in accordance to the manufacturer protocol. In brief, culture media was discarded from each well, while being carefully washed with PBS and stored on ice. The cells were then separated by gentle trypsinization and applying 0.5 ml of 0.5 x trypsin; afterwards they were re-suspended in the original media to insert the detached cells into the suspension for further examination. After exposing the 1.0×106 cells to 10 ml of binding reagent and 1.25 ml of FITC-labeled annexin V solutions, the cells were incubated in the dark for 15 min at room temperature. Then, the cell suspension was centrifuged at 1000 g for 5 min and then, the supernatant was removed and the cells were re-suspended in 0.5 ml of ice-cold 1X binding buffer. Finally, the cells were incubated with 10 ml of propidium iodide and were moved to FACS tubes (Fahrenheit, UK). The fluorescence data analysis was performed by WinMDI 2.8 software.

***Statistical analysis***

All data were expressed as mean±SD. Statistical analysis of the data was done via one-way ANOVA and *P-*Value<0.05 was selected as the significance level.

## Results

***Synthesis and characterization of synthesized vectors***

In this study, conjugation was performed via conjugation and physically interaction approaches. In the first conjugation method (І), peptide was conjugated to PEI using SPDP linker (І). In the second conjugation method (П), Conjugation of BR-2R-PEI was also done by EDC/NHS method. In the last conjugation method (ІП) the polymer was modified by the conjugation of 6-bromohexanoic acid to the amine groups of PEI and then BR-2R was conjugated to the terminal functional carboxylate groups using EDC/NHS method. Physical interaction approach (ІV) was done without any external linkage agent.

Using TNBS method, the extent of PEI primary amine substitution by 6-bromohexanoic, SPDP and BR-2R were calculated. The degree of substitution of 6-bromohexanoic was found to be (6%), SPDP was reacted with PEI in different molar ratios and results showed 0.66, 1.3, 1.8, and 4.39% of conjugation (Table 1). 

***Gel retardation assay***

**Figure 4:**
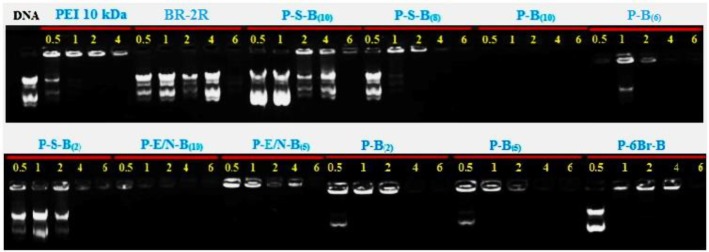
Gel retardation assay of polyplexes (PEI 10 kDa, BR-2R, P-S-B(10), P-S-B(8), P-B(10), P-B(6), P-S-B(2), P-E/N-B(10), P-E/N-B(5), P-B(2), P-B(5) and P-6Br-B) at different C/P ratios (0.5, 1, 2, 4, and 6)

To protect DNA from nucleases as well as increasing cellular uptake, complexation of pDNA and vector into nanoscale structures is necessary. In this regard, the rate of DNA mobility inhibition in agarose gel electrophoresis was evaluated. Polyplexes were prepared at various vector /DNA ratios and electrophoresed on agarose gel 1% (Figure 4). 

According to gel retardation images (Figure 4), except P-B(10) and P-B(6) vectors, other vectors were not able to condense pDNA at C/P ratio of 0.5. The gel image indicates that the P-E/N-B(10), P-B(5), P-B(10) and P-6Br-B vectors had ability to condense the pDNA at C/P ratio of 1. Also, except P-S-B(10) and PEI-6Br-B vectors, all vectors could condense pDNA at C/P ratio of 2. The condensation ability of all vector except P-S-B(10), P-E/N-B(5) and P-6Br-B vectors, are the same at C/P ratio of 4. All synthesized vectors including P-S-B(2), P-S-B(8), P-S-B(10), P-B(6), P-B(2), P-B(5), P-B(10), P-E/N-B(5), P-E/N-B(10), and P-6Br-B completely inhibited the migration of pDNA at C/P ratio of 6. Although the PEI 10 kDa had ability to condense the pDNA only at C/P ratio of 4 and 6 ratios, the BR-2R peptide alone could not able to condense the pDNA at any C/P ratio. 

***Polyplexes size and zeta potential***

The particle size of some synthesized vectors was in the range of 200-270 nm (Table 2). The particle size of unmodified PEI with plasmid was under 120 nm. In all synthesized vectors, the size increased compared to PEI. Also, in comparison with other conjugation approaches, the conjugation of PEI with BR-2R via EDC-NHS resulted in larger size vectors, while the non-linker based conjugation of PEI and BR-2R peptide resulted in the smaller polyplexes. However, P-B(5) polyplex had the smallest size (Table 2).

***Transfection efficiency of vectors***

**Figure 5:**
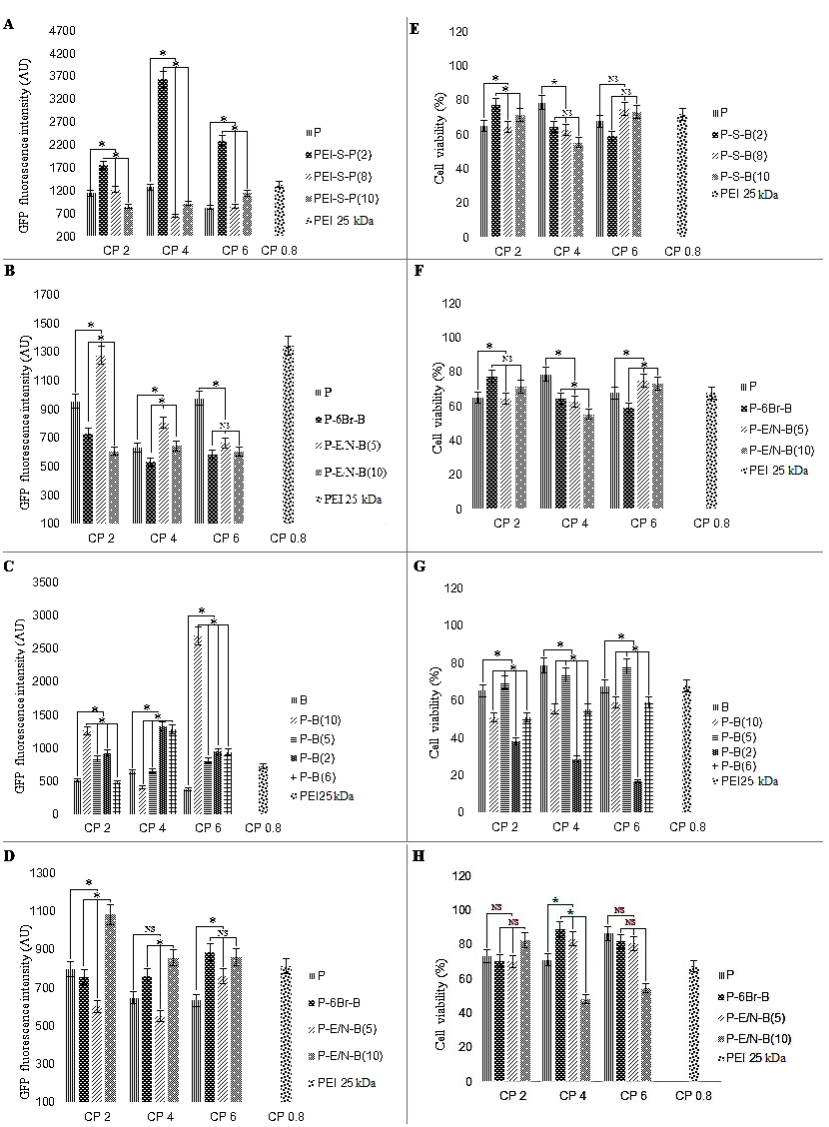
Transfection efficiency and cytotoxicity of polyplexes in HEK293 cell line (A-C and E-G), and L929 cell line (D and H). The green fluorescent protein (GFP)-based plasmid (pGFP) fluorescence intensity and cell viability are presented as the mean±standard deviation (SD) of triplicates. * *P*-value<0.05 and NS non-significant, modified PEI compared to unmodified PEI 25 kDa at C/P ratio of 0.8

**Figure 6:**
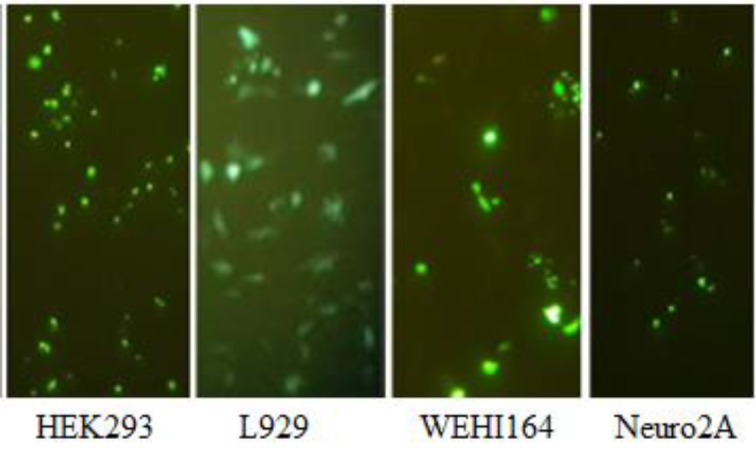
Transfection efficiency and cytotoxicity of polyplexes in cancer (WEHI164 (A and C) and Neuro2A cell lines (B and D)). The green fluorescent protein (GFP)-based plasmid (pGFP) fluorescence intensity (A and B) and cell viability (C and D) are presented as the mean ± standard deviation (SD) of riplicates. * *P*-Value<0.05 and NS non-significant, modified PEI compared to unmodified PEI 25 kDa at C/P ratio of 0.8

**Figure 7:**
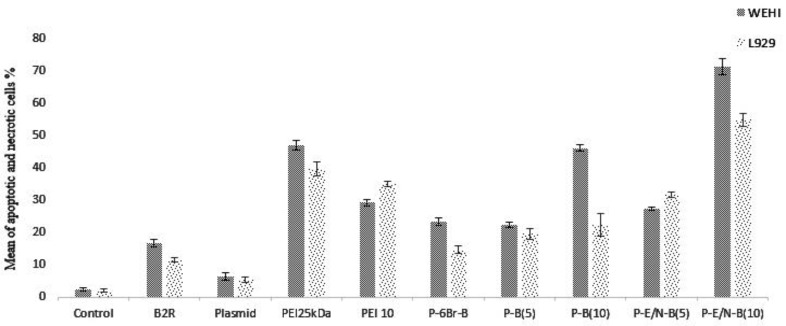
Expression of green fluorescent protein in HEK293, L929, WEHI164 and Neuro-2a cells transfected with polyplexes prepared from an EGFP expressing plasmid DNA and P-E/N-B(5) at C/P 4 that was most active in the transfection study

The transfection efficiency of modified PEI 10 kDa was investigated on HEK293 (as screening cell lines), L929 and WEHI164 (In order to access the vectors transfection efficiency on normal and cancer cell lines, respectively) and Neuro2A (to access the transfection efficiency of the best vectors in another cancer cell line) cell lines (Figures 5A-D and Figures 6A and B and Figure 7). The cell lines were transfected with various polyplexes with different ratios.

In compare to the non-modified polymer, among all polyplexes (which have been synthesized by SPDP, EDC-NHS, EDC-NHS+6-Bromohexannoinc acid and physical interaction approaches), the highest and lowest transfection efficiency has been observed for P-E/N-B(5) (at C/P ratio of 4) in Neuro2A cells (Figure 6B) and P-B(10) (at C/P ratio of 4) in HEK293 cell (Figure 5C), respectively. Also, among all synthesized vectors, the most effective transfection was regarded to the P-S-B(2), P-S-B(8) and P-S-B(10) vectors, (*P*<0.0001).

Also, among polyplexes which have been prepared by various methods, the highest transfection efficiency has been observed for P-S-B(2) (at C/P ratio of 4), P-E/N-B(10) (at C/P ratio of 2), P-E/N-B(5) (at C/P ratio of 6) and P-E/N-B(5) (at C/P ratio of 4), for HEK293, L929, WEHI164 and Neuro2A cell line, respectively (Figures 5A-D and Figures 6A and B).

***Cell viability of vectors***

The cell viability of modified PEI 10 kDa against HEK293, L929, WEHI164 and Neuro2A cell lines was carried out using MTT assay. The polyplexes were prepared at the same C/P ratios used in transfection experiment. Branched 25 kDa PEI was used as control at C/P ratio of 0.8. 

As shown in Figures 5 and 6, the P-6Br-B vector (C/P~6) had the highest metabolic activity in L929 cells (Figure 5H). In compare to this vector, the highest cytotoxicity rate has been observed for P-S-B(2) vector (C/P~4) in Neuro2A cells (Figure 6D).

Among the vectors, the highest metabolic activity had been observed for P-B(5) (C/P~6), P-6Br-B (C/P~6), P-6Br-B (C/P~4) and P-6Br-B (C/P~4), for HEK293, L929, WEHI164 and Neuro2A cells, respectively (Figures 5E-H and Figures 6C and D).

***Apoptosis assay results***

**Figure 8:**
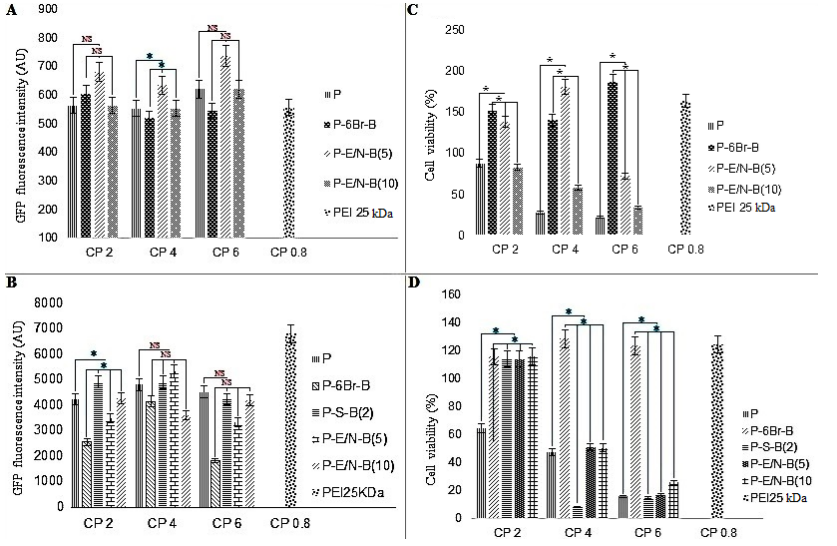
Apoptotic effects of the most effective transfected vectors on L929 and WEHI164 cells at C/P ratio of 4. The assay was carried out by PI/Annexin V method. Each column represents the average of three independent experiments

**Figure 9:**
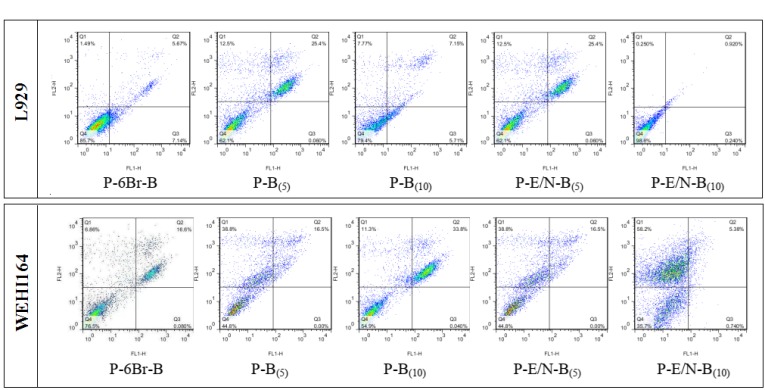
Representative dot plots of PI/annexin V staining of L929 and WEHI164 cells (upper-right quadrant: cells in the late stage of apoptosis, upper-left quadrant: dead cells, lower-right quadrant: cells undergoing apoptosis, lower-left quadrant: viable cells)

The apoptotic effects of synthesized vectors were carried out by PI/Annexin V assay on WEHI164 and L929 cell lines. The results showed that the highest apoptosis rate occurred in the presence of P-E/N-B(10) vector for WEHI164 cells (Figure 8). The apoptosis results of the most effective vectors, indicated that these vectors had the highest apoptosis induction ability on WEHI164. In compared to WEHI164 as cancer cell, these vectors had less apoptotic induction ability on L929 as normal cell (Figure 9). 

## Discussion

Gene therapy can be broadly defined as the defined genetic material transferring into the specific patient’s cell for preventing or altering a particular disease state and even killing the patient’s cell. To this end, the gene must successfully overcome the extracellular and intracellular barriers. In general, viral carriers (29), non-viral carriers (30), and physical methods (31) are the main approaches that have been used for gene delivery. Since non-viral carriers do not show a comparable transfection efficiency to viral vectors, it is quite necessary to exploit the capability of CPPs to enhance the cellular penetration of non-viral carriers. The positive charges of these CPPs can increase the chances of penetration via electrostatic interaction with negative charges compartment of cell membrane (32).

PEI is one of the non-viral carriers that has been studied extensively for *in vivo* and *in vitro* gene delivery, since 1995 (33). Its relatively high transfection efficiency seems to be attributed to its endosomal escape capability (presence of proton sponge effect) (34). 

**Table 1:** Prepared vectors by conjugation approaches (using SPDP, EDC/NHS and EDC/NHS+6-bromohexanoic acid) and physical interaction method

Vector name	Abbreviation	Synthesis protocol	Initial peptide/polymer ratio	Calculated peptide/ polymer
PEI (10 kDa)-SPDP*-Brevinin 2-R	P-S-B_(2)_	SPDP	1:2	1.66
PEI (10 kDa)-SPDP-Brevinin 2-R	P-S-B_(5)_	SPDP	1:5	1.13
PEI (10 kDa)-SPDP-Brevinin 2-R	P-S-B_(8)_	SPDP	1:8	1.5
PEI (10 kDa)-SPDP-Brevinin 2-R	P-S-B_(10)_	SPDP	1:10	4.39
PEI (10 kDa)- [(EDC**)-(NHS***)]-Brevinin 2-R	P-E/N-B_(5)_	EDC-NHS	1:5	6.5
PEI (10 kDa)-[(EDC)-(NHS)]-Brevinin 2-R	P-E/N-B_(10)_	EDC-NHS	1:10	9
PEI (10 kDa)-(6-Bromohexanoic acid)-Brevinin 2-R	P-6Br-B	EDC-NHS+6-bromohexanoic acid	1:5	6
PEI (10 kDa)-Brevinin 2-R	P-B_(2)_	Physical interaction	1:2	1.9
PEI (10 kDa)-Brevinin 2-R	P-B_(5)_	Physical interaction	1:5	2.3
PEI (10 kDa)-Brevinin 2-R	P-B_(6)_	Physical interaction	1:6	2.8
PEI (10 kDa)-Brevinin 2-R	P-B_(10)_	Physical interaction	1:10	4.81

*(Succinimidyl 3-(2-pyridyldithio) propionate, **(1-Ethyl-3-(3-dimethylaminopropyl)-carbodiimide, and *** (N-hydroxysuccinimide)

**Table 2:** Mean size and zeta potential of polyplexes at vector/DNA weight ratio of 4 (mean ± standard deviation, n=3)

Name	Polyplexes size (nm)	Polydispersity index (PDI)	Polyplexes zeta potential (mv)
PEI 10 kDa	109.3	0.44±0.04	12.7±1.12
BR-2R	685.0	0.64±0.06	-9.42±0.92
P-S-B_(2)_	163. 6	0.35±0.01	26.9±1.22
P-E/N-B_(10)_	250.6	0.48±0.02	21.5±1.76
P-B_(2)_	212.6	0.45±0.01	23.8±1.57
P-B_(5)_	121.8	0.38±0.07	28.4±1.19
P-B_(10)_	190.1	0.41±0.01	24.6±1.98

Besides to these benefits, PEI-based gene delivery suffers from some related toxicity (35-39). Therefore, the main purpose of this study was to design a hybrid vector that are composed of PEI 10 kDa and BR-2R in such a way that it can produce less toxicity while maintaining the high transfection efficiency.

As it has been noted in previous studies, when the molecular weight of PEI is reduced (the same as our study), the size of the resulting polyplex increases. Polyplexes with larger size have some advantages along with disadvantages. Although the condensed DNA does not have sufficient compaction in the structure of polyplexes that have large sizes, yet it can be released much easier than smaller polyplexes. On the other hand, as the size of a polyplex increases, its potential for penetrating into a cell will be decreased (40).

In our study, we used BR-2R as a new cell penetrating peptide, in order to enhance cellular uptake and reducing the effects of size on the transfection efficiency.

In order to conjugate BR-2R peptide to PEI 10 kDa, two different approaches have been used throughout this project (Conjugation with and without linker). At the first approach, we applied the SPDP hetero-functional cross-linker and 6-bromohexanoic acid linker. In another method, the non-linker based approach has been employed to conjugate the BR-2R peptide to the PEI. The main reason for using two different functionalization approaches was to compare synthesis protocols and also evaluate the transfection efficiency of synthesized vector.

The results of this study show that the synthesized vectors had the size in the range of 200-270 nm (Table 2). In comparison to physical interaction method the results showed that the conjugation approaches resulted in smaller size vectors. Although cellular uptake of these small vectors can be more significant than the larger vectors, but this small size may be a physical barrier for pDNA uploading and releasing from polyplex (41).

The ability of a polyplex in the gene condensation is a critical factor for gene therapy. The results of the gel retardation assay showed that the P-B(5), P-B(10) and P-E/N-B(10) polyplexes (at C/P ratio of 2, 4 and 6) are completely able to condense pDNA (Figure 4). Actually, this condensation phenomenon is the result of the electrostatic interactions between negative pDNA charges and positive charges of PEI 10 kDa. Therefore, the low positive charges on PEI 10 kDa surface will be a key factor in decreasing the condensation efficiency of polyplexes. As expected, in all C/P ratios, the BR-2R peptide has not been able to condense pDNA. Compared to all synthesized vector, the P-S-B(10) vectors in different ratios of C/P did not have sufficient ability to condense the pDNA (Figure 4). In compared to the physical interaction method, as the SPDP mediated peptide linkage give rise to forming more compacted polyplexes and smaller vector size, it may reduce complete condensation of pDNA by steric hindrance. Another reason for the decreasing the condensation ability of vectors prepared by conjugation approaches could be the decrease in the positive charge on the surface of PEI 10 kDa.

The size range of the synthesized vectors reflects this fact that most of these vectors may uptaked by cells via the macropinocytosis. In this process the vectors are surrounded by cell membrane and enter into the cell via macropinosome vesicle (42, 43) Moreover, if the uptake of the vectors happens via endocytosis, there are two obstacles (macropinosome and endosome) that should be removed. In this study, it seems that the BR-2R peptide could acts as a CPP and enhances the cellular uptake of polyplexes (34).

The results of this study indicate that the most efficient synthetic vector for transfection is P-E/N-B(5) (C/P~4), which has the highest transfection for Neuro2A cells compared to other cell lines (HEK293, L929 and WEHI164) (Figure 6B). In other words, in compared to other conjugation methods, the non-linker based conjugation method showed the best results. The reason could be the faster dissociation of the polymer-peptide complex after its release from endosome, which is due to the poor physical connection between the BR-2R peptide and PEI 10 kDa. Some studies showed that the BR-2R peptide have more specificity for cancer cell rather than normal cells (21). Taking these points into account, it is expected that the transfection efficiency of BR-2R peptide containing vector in normal cell be lower than cancerous cell lines. Our results showed that the lowest transfection rate is regarded to P-B(10) for HEK293 (as normal cell line) (Figure 5C).

The lowest toxicity was observed for P-6Br-B vector (at C/P ratio of 4), which causing the highest metabolic activity in L929 cells compared to HEK293, WEHI164 and Neuro2A cell lines (Figure 5H). This high metabolic activity is due to lower penetration affinity of this vector in the presence of BR-2R peptide to the normal cells (such as L929). In compared to P-6Br-B vector, the P-S-B(2) vector (at C/P ratio of 4) have the lowest metabolic activity for Neuro2A cells compared to the HEK293, L929, and WEHI164 cell lines (Figure 6D). The reason for this toxicity will be due to the high specificity of BR-2R peptide binding to the cancerous cell in compared to normal cell.

Besides to more specificity of BR-2R peptides to cancerous cells, some researcher showed that this peptide also has the apoptosis induction effects. Actually the highest apoptosis rate occurred in the presence of P-E/N-B(10) vector in WEHI164 cells (Figure 8). 

BR-2R peptide has increased the transfection and also the cytotoxicity in cancerous cells in compared to normal cells. The low transfection efficiency of BR-2R for normal cell could be attributed to its less specificity to normal cells. PEI 10 kDa has strong ability for condensing pDNA in most C/P ratio. In compared to physically synthesized vectors, the vectors which were synthesized by conjugation approaches (including SPDP, EDC/NHS and EDC/NHS+6-bromohexanoic acid) have less ability in pDNA condensation in most C/P ratio.

## Conclusion

It could be concluded that with conjugation of BR-2R peptide to the polyethyleneimine, not only the transfection efficiency of synthesized vectors has improved on the normal cells but due to their significant apoptosis properties on the cancer cells, these vector can be considered as a bright promising vectors for future cancer treatment.
